# Dietary camellia seed oil attenuates liver injury in mice chronically exposed to alcohol

**DOI:** 10.3389/fnut.2022.1026740

**Published:** 2022-10-11

**Authors:** Rui Guo, Jinyan Zhu, Lin Chen, Jiaomei Li, Qinchao Ding, Qiang Han, Weijun Zheng, Songtao Li

**Affiliations:** ^1^School of Public Health, Zhejiang Chinese Medical University, Hangzhou, China; ^2^Academy of Chinese Medical Science, Zhejiang Chinese Medical University, Hangzhou, China

**Keywords:** dietary fat, alcoholic liver disease, monounsaturated fatty acids, camellia seed oil, liver injury, Lieber-DeCarli diet

## Abstract

Dietary fat composition is closely associated with the pathological development of alcoholic liver disease (ALD). Fat enriched with saturated fatty acids protects whereas with polyunsaturated fatty acids aggravates alcohol-induced liver injury. However, limited study has addressed how monounsaturated fatty acids (MUFAs) determines the pathological process of ALD. Our study was conducted to evaluate the effect of MUFAs-enriched-camellia seed oil (CSO) on alcohol-induced liver injury. The ALD model was established by feeding C57BL/6 mice with Lieber-DeCarli diet, and with either CSO or polyunsaturated fatty acids (PUFAs)-enriched-corn oil (CO) as fat source. After 4-week-intervention, CSO-feed rescued alcohol-induced liver injury compared to CO-feed, evidenced by measurements of plasma ALT activity, H&E stain, and hepatic cleaved-Caspase-3 expression. Besides, CSO-feed alleviated alcohol-induced oxidative stress, associated with NRF2 and Hif-1α expressions improvement. The reduction of F4/80 immunostaining and the decreased expressions of hepatic TNF-α and IL-6 suggested CSO-feed improved alcohol-induced inflammation. The mechanistic analysis showed that the inhibition of ASK1 and MAPKs might contribute to CSO-protected liver injury. Notably, we observed CSO-feed relieved the gut microbiota disturbance with the decreased *Firmicutes* and *Turicibater*, and the increased *Bacteroidota, Alloprevotella*, and *Bacteroides*, and reduced circulatory endotoxin level and lipolysis of adipose tissue, which are the known pathogenic factors in alcohol-induced liver injury. Unexpectedly, CSO induced more hepatic steatosis than CO-feed. In conclusion, CSO attenuated chronic alcohol consumption-induced liver injury but enhanced hepatic steatosis. CSO could be a potential dietary choice for alcoholic individuals with liver injury.

## Introduction

The harmful use of alcohol has been a worldwide problem. It is estimated that alcohol abuse represents the world's third largest risk factor for disease and disability (1). Liver is the main organ responsible for metabolizing alcohol, which has been considered for long time the major victim of the harmful use of alcohol, with much of the burden depending on alcoholic liver disease (ALD). Due to its significant morbidity, mortality, and burden on the health care system, ALD has become a serious public health problem with global ramifications (2). Unfortunately, few satisfactory advances have been made in management of ALD until now, except abstinence from alcohol. Thus, novel and more practical treatment options are urgently needed.

Epidemiological studies indicate that nutritional modulation of ALD is an attractive approach, and the combined effect of dietary fat and alcohol consumption on ALD has been increasingly investigated (3, 4). Several studies already indicated that diets enriched in saturated fatty acids or n-3 polyunsaturated fatty acids, such as cocoa butter, lard, or fish oil, protected against alcohol-induced liver injury, whereas diets enriched in n-6 polyunsaturated fatty acids (PUFAs), such as corn oil (CO), exacerbated liver dysfunction by using either the Lieber-DeCarli liquid diet or the intragastric alcohol-fed animal model (5–8). Up to now, studies on the effect of dietary fat on ALD have mainly focused on saturated and polyunsaturated fatty acids. However, limited study has addressed how monounsaturated fatty acids (MUFAs) determines the pathological process of ALD.

Camellia seed oil (CSO), one of editable high-quality oils recommended by Food and Agriculture Organization, is known for a wide spectrum of applications and therefore has become the focus of some research attention over recent decades. CSO is a MUFA enriched plant-derived oil, including about 80% oleic acid (OA, C18:1n-9) (9). Accumulating evidence showed the beneficial effects of CSO on the improvement of metabolic liver diseases in recent years. Pretreatment of male SD rats with CSO (150 g/kg diet) suppressed CCl_4_-induced hepatic oxidative injury (10). Besides, oral administration of CSO alleviated high fat diet-induced liver dysfunction via rescuing gut microbiota disorder (11, 12), which is also a pathogenic factor to ALD (13, 14). CSO intervention for 24 days significantly ameliorated acetic acid-induced colitis in rats by slightly enhancing antioxidant enzymes activities and significantly reducing inflammatory damage (15). However, little studies have been conducted to explore the protective role of CSO on chronic alcohol consumption-induced liver injury.

Therefore, in the present study, we aimed to evaluate the biological effect of MUFAs enriched CSO supplementation as unique dietary fat source on chronic alcohol-induced liver dysfunction in C57BL/6 mice. Consistent with our expectation, CSO supplementation rescued liver injury, hepatic oxidative stress, and inflammation in response to alcohol. Unexpectedly, CSO administration enhanced alcohol-induced hepatic steatosis. The present results provide preliminary insights for dietary CSO in the pathogenesis of ALD and CSO could be recommended for alcoholic individuals with liver injury, oxidative stress, and inflammation being the principal pathological mechanisms.

## Materials and methods

### Materials and reagents

The traditional Lieber-DeCarli alcohol liquid diet and an isocaloric control diet were purchased from Trophic Animal Feed High-tech Co., Ltd. (Nantong, China). Commercial assay kits for alanine aminotransferase (ALT), aspartate transaminase (AST), free fatty acid (FFA), glycerol, malondialdehyde (MDA), and Glutathione Peroxidase (GSH-PX) were purchased from Nanjing Jiancheng Bioengineering Research Institute (Nanjing, China). Triglyceride (TG) assay kit and total cholesterol (TC) assay kit were purchased from Applygen Technologies Inc. (Beijing, China). Oil Red O solution and haematoxylin and eosin (H&E) staining were obtained from Beijing Solarbio & Technology Co., Ltd. All other regular reagents were of the highest analytical purity.

### Animals experimental design and sample preparation

Twenty-four SPF male C57BL/6 mice (8-week-old) were purchased from the Shanghai Sippe-Bk Lab Animal Co., Ltd, Shanghai, China. Mice were kept in an environment of 55 ± 5% relative humidity, 23 ± 2°C, and 12 h light and dark cycles. After 1-week acclimatization, mice were divided into six animals per group at random and different experimental diets were fed for four weeks as follows: (a) pair-fed (PF) with corn oil (CO) group (PF/CO), mice were fed Lieber-DeCarli CO liquid diets containing isocaloric maltose dextrin as CO control; (b) alcohol-fed (AF) with CO group (AF/CO), mice were fed alcohol-containing modified Lieber-DeCarli CO liquid diets; (c) PF with camellia seed oil (CSO) group (PF/CSO), mice were fed Lieber-DeCarli CSO liquid diets containing isocaloric maltose dextrin as CSO control; (d) AF with CSO group (AF/CSO), mice were fed alcohol-containing modified Lieber-DeCarli CSO liquid diets. The components of liquid diets and exposure time were shown in Table 1. CO and CSO were bought from the local market. The fatty acid composition of CSO and CO were provided in Table 2. Liquid diets were freshly prepared from powder daily according to the manufacturer's instruction. Average daily caloric of liquid intake per mouse was monitored and calculated in Figure S1. After 12 h of fasting, mice were anesthetized with pentobarbital solution (50 mg/kg body weight) (16). Livers and adiposes were dissected quickly, washed twice with saline, blotted dry on a filter paper, and their weights were measured. The livers, adiposes, blood samples and colonic stool were collected and stored at −80°C for further analysis.

**Table 1:** The components of liquid diets.

**Group**	**Day**	**Alcohol**	**Carbohydrate**	**Fat**	**Protein**
				**(CO or CSO)**	
PF	Week 1–4	0.0%	47%	35%	18%
AF	Day 1–3	0.0%	47%	35%	18%
	Day 4–5	5.5%	41.5%		
	Day 6–7	11.0%	36%		
	Week 2	22.0%	25%		
	Week 3	27.0%	20%		
	Week 4	32.0%	15%		

CO, corn oil; CSO, camellia seed oil; PF, pair-fed; AF, alcohol-fed.

**Table 2:** Fatty acid composition of dietary fats.

**Fatty acid composition (g/100 g**	**Corn oil**	**Camellia seed oil**
**of total fatty acid)^a^**		
C16:0	12.47 ± 0.13	9.41 ± 0.06
C18:0	1.72 ± 0.04	2.02 ±0.03
C18:1	26.54 ± 0.08	78.37 ± 0.11
C18:2	59.27 ± 0.25	10.20 ± 0.01
Ratio of 18:1/18:2	0.45	7.68
Ratio of UFAs to SFAs	6.06	7.75

UFAs, unsaturated fatty acids; SFAs, saturated fatty acids.

a Fatty acid composition was determined by gas chromatography.

### Cell culture and treatments

AML-12 cells were incubated in DMEM/F12 containing 10% (v/v) fetal bovine serum (FBS), 10 μg/ml insulin, 5 μg/ml transferrin, 5 ng/ml selenium (Sigma-Aldrich, St. Louis, MO), and 40 ng/ml dexamethasone (Sigma-Aldrich, St. Louis, MO) at 37°C under 95% air and 5% CO_2_ atmosphere. AML-12 cells were treated with 100 μM linoleic acid (LA, Sigma-Aldrich, St. Louis, MO, O5507) and 100 μM oleic acid (OA, Sigma-Aldrich, St. Louis, MO, O1008) with/without 200 mM alcohol (Merck KGaA, Darmstadt, Germany) for 16 h. The lipid deposition of AML-12 cells was detected by measuring the TG content via commercial assay kit.

### Histological examination

Livers were fixed in a 4% buffered paraformaldehyde (Biosharp Biotechnology, Shanghai, China) for 48 h. Liver tissues were embedded with an optimum cutting temperature (OCT) embedding agent and sliced on a cryostat. The 4 μm-thick liver sections were stained with H&E solution. The 8 μm-thick frozen sections were stained with Oil Red O solution.

### Immunofluorescence analysis

The frozen liver sections (8 μm) were incubated in blocking solution and subsequently incubated with fluorescent antibodies to detect F4/80 macrophages (CST, 30325S). Alexa fluor 647 (Dawen Biotec, WBA0468) was used as a secondary antibody.

### Biochemical analysis

Blood samples from inferior vena cava were centrifuged at 3,000 rpm for 15 min, and the supernatants were collected for measurement of the activity of ALT, and the levels of TG, TC, FFA and glycerol. Liver samples (~20 mg) were homogenized using a homogenizer and then were centrifuged at 3,000 rpm for 15 min. Afterwards, the hepatic levels of TG, TC, MDA and GSH-PX were also measured by commercially available kits.

### 16S rRNA sequencing

Colonic stool was directly collected from extracted colon and frozen at −80°C. Total microbial genomic DNA was extracted using the E.Z.N.A.^®^ DNA Kit (Omega Bio-tek, Norcross, GA) according to manufacturer's instructions. The hypervariable region V3–V4 of the bacterial 16S rRNA gene were amplified with primer pairs 341F (5′-CCTAYGGGRBGCASCAG-3′) and 806R (5′-GGACTACHVGGGTWTCTAAT-3′). Purified amplicons were pooled in equimolar amounts and paired-end sequenced on an Illumina NovaSeq PE250 platform (Illumina, San Diego) according to the standard protocols by Majorbio Bio-Pharm Technology Co. Ltd. (Shanghai, China). The raw sequencing reads were deposited into the NCBI Sequence Read Archive (SRA) database (Accession Number: SUB11510319). The taxonomy of each OTU representative sequence was analyzed by RDP Classifier version 2.2 against the 16S rRNA gene database using confidence threshold of 0.7. Bioinformatic analysis of the gut microbiota was carried out using the Majorbio Cloud platform.

### Western-blot analysis

Phenylmethanesulfonyl fluoride (PMSF), radioimmunoprecipitation assay (RIPA) lysis buffer, and the sodium dodecyl sulfate polyacrylamide gel electrophoresis (SDS–PAGE) gel preparation kit were purchased from Beyotime (Shanghai, China). Liver and adipose tissues were treated with PMSF and RIPA to extract total protein. The lysate was incubated on ice for 30 min and then centrifuged at 12,000 rpm for 15 min. Moreover, a nuclear extraction kit (Keygen Biotech Corp.Ltd. Jiangsu, China) was used for some special protein. Polyvinylidene fluoride (PVDF) membranes were supplied from Millipore (Burlington, MA). Protein samples (~30 μg) were separated by SDS–PAGE and transferred to PVDF membranes. The concentrations of proteins were determined by a bicinchoninic acid (BCA) assay kit (Beyotime, China). The following antibodies were used for western-blot: anti-cleaved-Caspase 3 (CST, 9661S), anti-HIF-1α (CST, 36169S), anti-NRF2 (Abcam, ab137550), anti-4-HNE (Abcam, ab46545), anti-TNF-α (Proteintech Group, 60291-1-Ig), anti-IL-6 (Proteintech Group 66146-1-Ig), anti-IL-1β (ImmunoWay, YT2322), anti-TLR4 (Santa Cruz, sc-293072), anti-MYD88 (CST, 4283S), anti-phospho-ASK1 (Santa Cruz, sc-166967), anti-ASK1 (CST, D11C9), anti-phospho-JNK (Bioss, bs-1640R), anti-JNK (CST, 3708S), anti-phospho-p38 (CST, 4511S), anti-p38 (CST, 9212S), anti-phospho-ERK1/2 (CST, 4370S), anti-ERK1/2 (CST, 4695S), anti-SREBP-1c (Santa Cruz, sc-365513), anti-CD36 (Santa Cruz, sc-7309), anti-FATP2 (Proteintech Group, 14048-1-AP), anti-DGAT2 (Santa Cruz, sc-293211), anti-ATGL (Santa Cruz, sc-365278), anti-HSL (Santa Cruz, sc-74489), anti-VLDLR (Proteintech Group, 19493-1-AP), anti-β-actin (ABclonal, AC026) and anti-Histone H3 (Huabio,EM30605). β-actin and Histone H3 were used as an internal control. The secondary antibodies were anti-rabbit (Boster Biological Technology, ba1054) and anti-mouse (Boster Biological Technology, ba1050). Bands were quantified by Image J software.

### Statistical analysis

All data were analyzed using GraphPad Prism 9.00 software (GraphPad Software, CA). Results were represented as mean ± standard deviation (SD). Two-tailed Student *t* tests were used to compare two groups, and the one-way analysis of variance (ANOVA), followed by the Turkey multiple-comparison test, was used to determine multiple groups. *P* < 0.05 was considered to represent statistical significance.

## Results

### Dietary CSO supplementation rescues alcohol-induced liver injury

**Figure 1:**
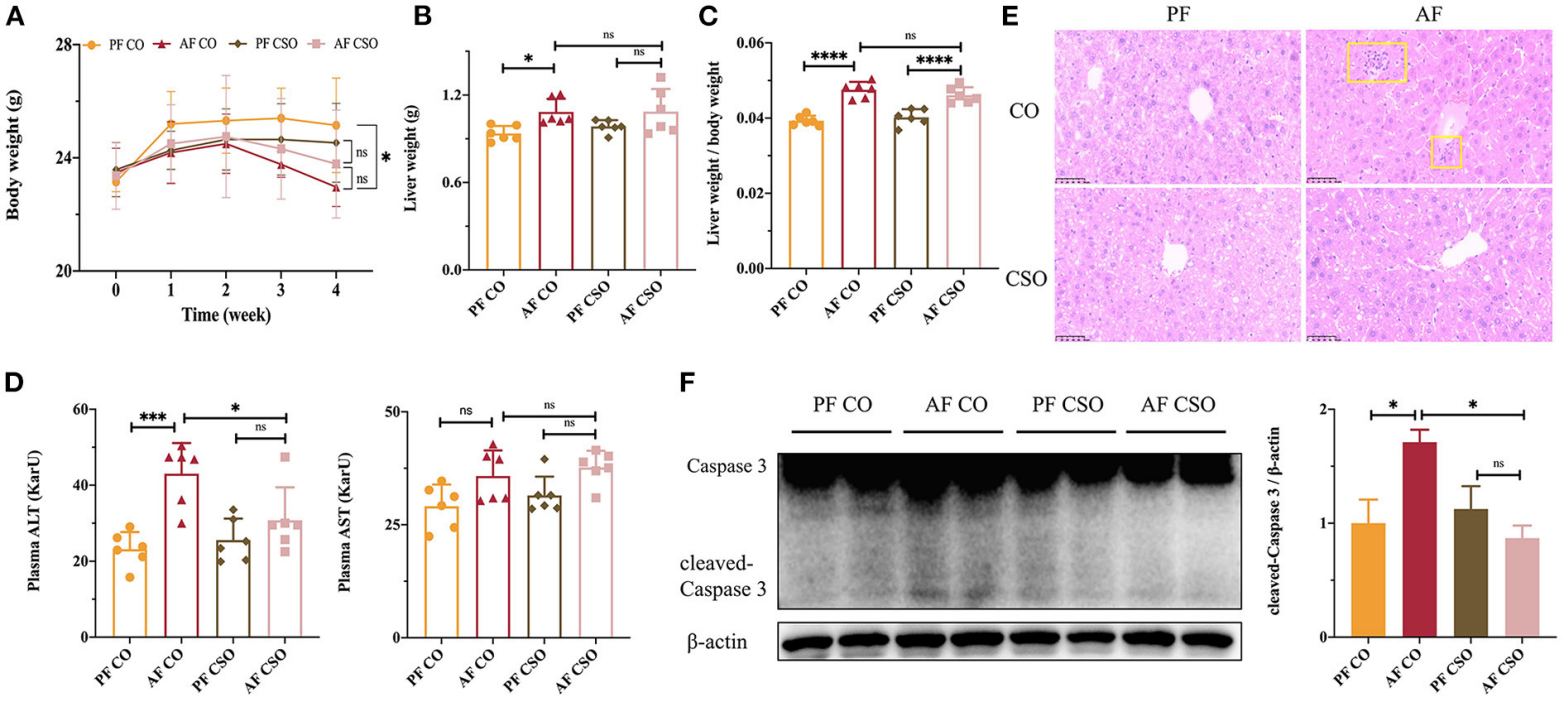
Dietary CSO supplementation rescues alcohol-induced liver injury. **(A)** The body weight. **(B)** The liver weight. **(C)** The ratio of liver-to-body weight. **(D)** The activities of plasma ALT and AST. **(E)** H&E staining (400×). **(F)** The expression of hepatic cleaved-Caspase 3. *P < 0.05, ***P < 0.001, and ****P < 0.0001 indicate statistically significant.

Initially, there was no significant difference in body weight (BW) among four groups. After 4 weeks feeding, the final BW in AF/CO group was obviously declined compared with that in PF/CO group; however, there was no difference in BW between the AF/CSO and PF/CSO groups (Figure 1A). The liver weight (LW) in AF/CO group was significantly increased compared with that in PF/CO group. However, the difference in LW between AF/CSO and PF/CSO groups was not statistically significant (Figure 1B). Moreover, the ratio of liver-to-body weight in AF group regardless of dietary fat was significantly elevated compared with that in PF group (Figure 1C). Furthermore, the activity of plasma ALT in AF/CO group was obviously increased by 1.86-fold compared with that in PF/CO group. The elevation of ALT induced by alcohol was effectively suppressed by dietary CSO supplementation, while there was no significant difference in AST level among these four groups (Figure 1D). We also observed pathophysiological changes in mice livers of diverse groups by H&E staining, and found that dietary rich in CSO significantly alleviated alcohol-induced hepatic histopathological injury (Figure 1E). Additionally, compared with CO, the elevated hepatic protein level of cleaved-Caspase 3 induced by alcohol was rescued by dietary CSO supplementation (Figure 1F).

### Dietary CSO supplementation alleviates alcohol-induced hepatic oxidative stress

**Figure 2:**
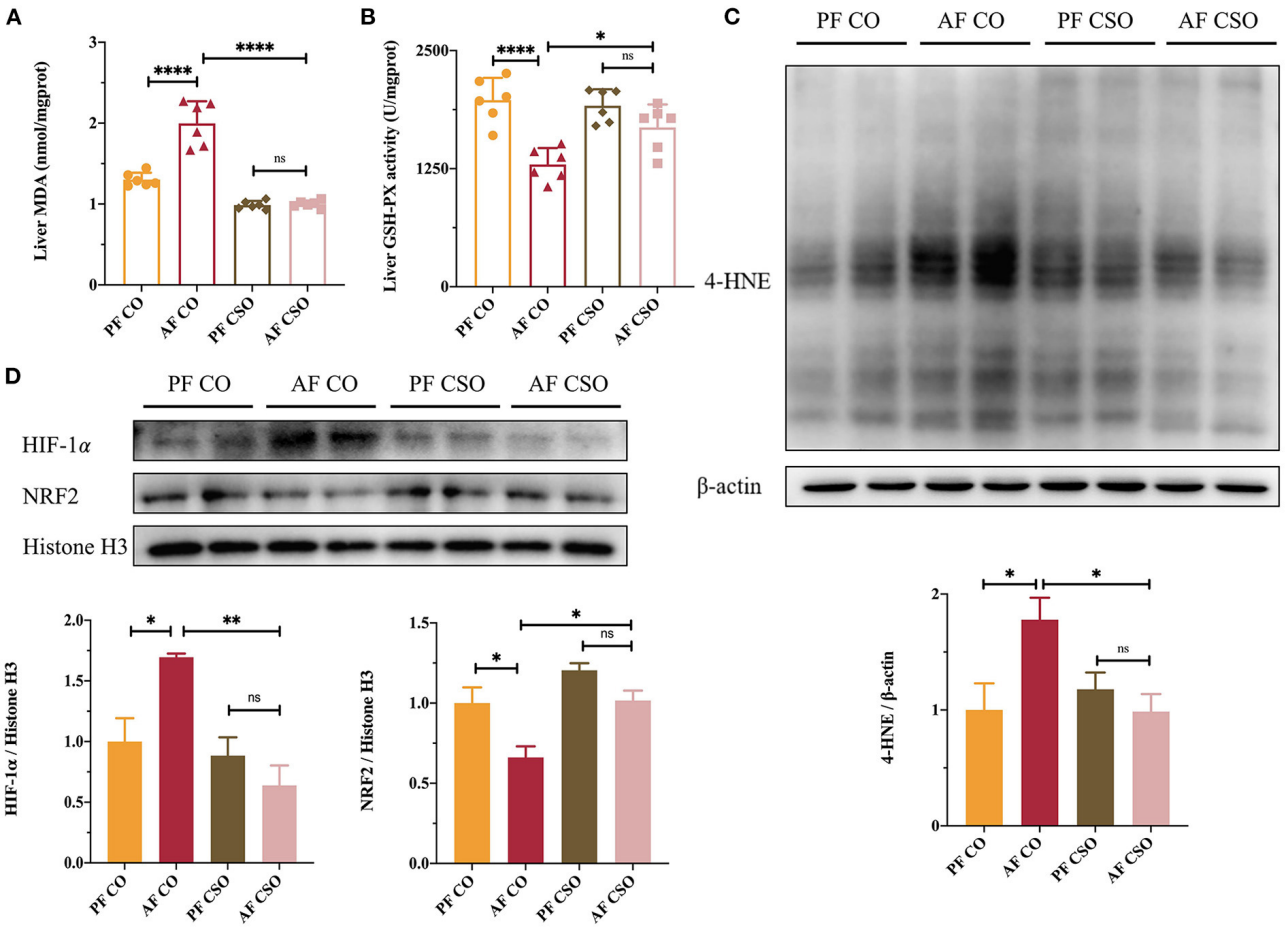
Dietary CSO supplementation alleviates alcohol-induced hepatic oxidative stress. The activity of **(A)** MDA and the level of **(B)** GSH-PX in livers. **(C)** The expression of hepatic 4-HNE. **(D)** The nucleoprotein expressions of hepatic HIF-1α and NRF2. *P < 0.05, **P < 0.01, and ****P < 0.0001 indicate statistically significant differences.

As shown in Figures 2A,B, the MDA activity was significantly increased and the GSH-PX level was obviously decreased in the liver tissues of AF/CO group compared with that in PF/CO group or AF/CSO group, suggesting that dietary CSO supplementation could decrease the hepatic MDA activity and improve the hepatic GSH-PX level in mice with alcohol-induced hepatic oxidative stress. In parallel, 4-HNE is also a critical lipid peroxidation product induced by oxidative stress and plays a dominant role in alcohol-induced liver injury (17). Compared with AF/CO group, dietary rich in CSO apparently decreased the elevated expression of 4-HNE induced by 4-week alcohol feeding (Figure 2C). Additionally, the expression of HIF-1α protein in AF/CSO group was significantly decreased and the expression of NRF2 protein in AF/CSO group was obviously increased compared with AF/CO group, demonstrating that dietary CSO could enhance the oxidant defense system by downregulating HIF-1α protein and upregulating NRF2 protein (Figure 2D).

### Dietary CSO supplementation improves alcohol-induced liver inflammation

**Figure 3:**
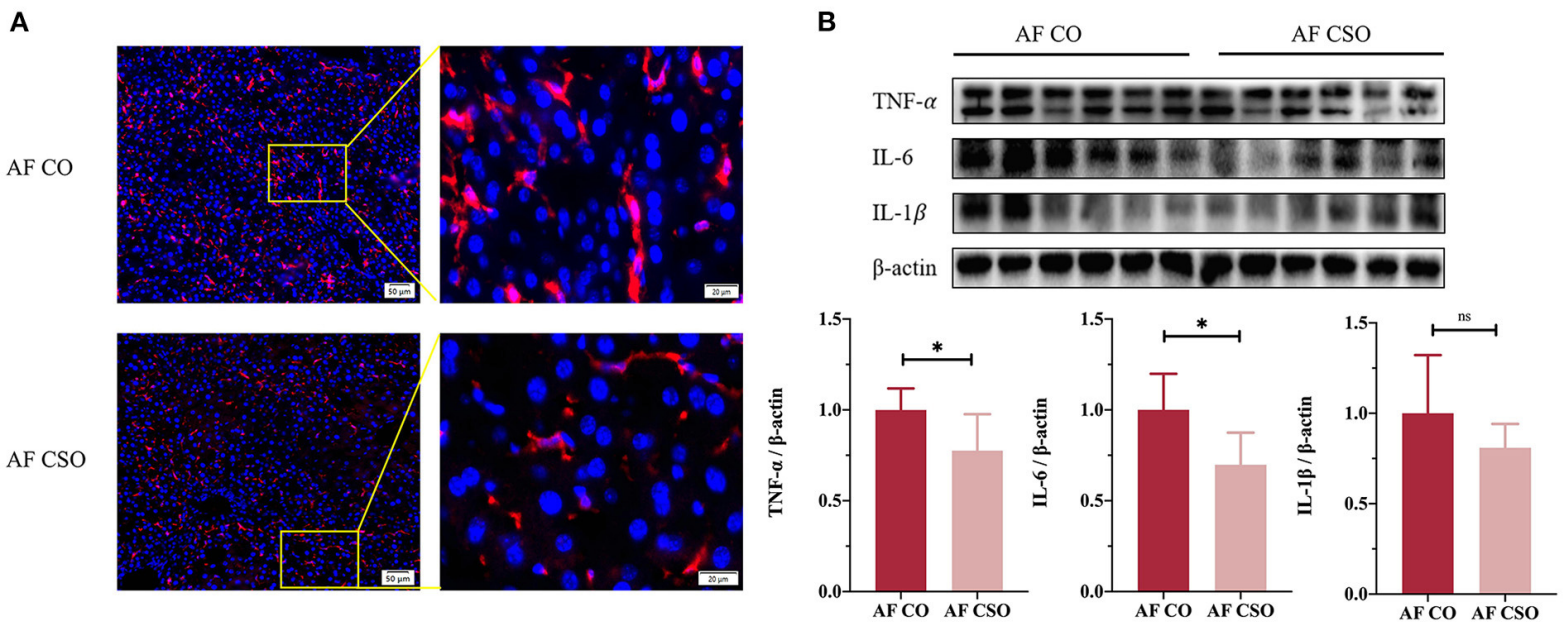
Dietary CSO supplementation improves alcohol-induced liver inflammation. **(A)** Representative immunofluorescent staining of F4/80 in mouse liver sections. Higher magnification of the indicated area (box) was shown in right. Red = F4/80, blue = DAPI (nuclei). Scale bars, 50 (left) and 20 (right) μM. **(B)** Protein maps and quantitative plots of TNF-α, IL-6, and IL-1β in mice livers. *P < 0.05 indicates statistically significant differences.

To assess the role of dietary CSO supplementation in alcohol-induced liver inflammation, we first performed immunofluorescence analysis of F4/80 (a marker for macrophage/Kupffer cell). The reduction of F4/80 immunostaining was detected in AF/CSO group compared to the AF/CO group (Figure 3A). Additionally, pro-inflammatory cytokines including TNF-α and IL-6 in mice livers were significantly decreased in the AF/CSO group compared to the AF/CO group; however, the difference in the expression of IL-1β between AF/CO and AF/CSO groups was not statistically significant (Figure 3B). It showed that dietary CSO supplementation could exert anti-inflammatory effect in liver tissues of mice with alcohol-induced liver injury.

### ASK1/MAPKs signaling pathways are involved in CSO-protected liver injury

**Figure 4:**
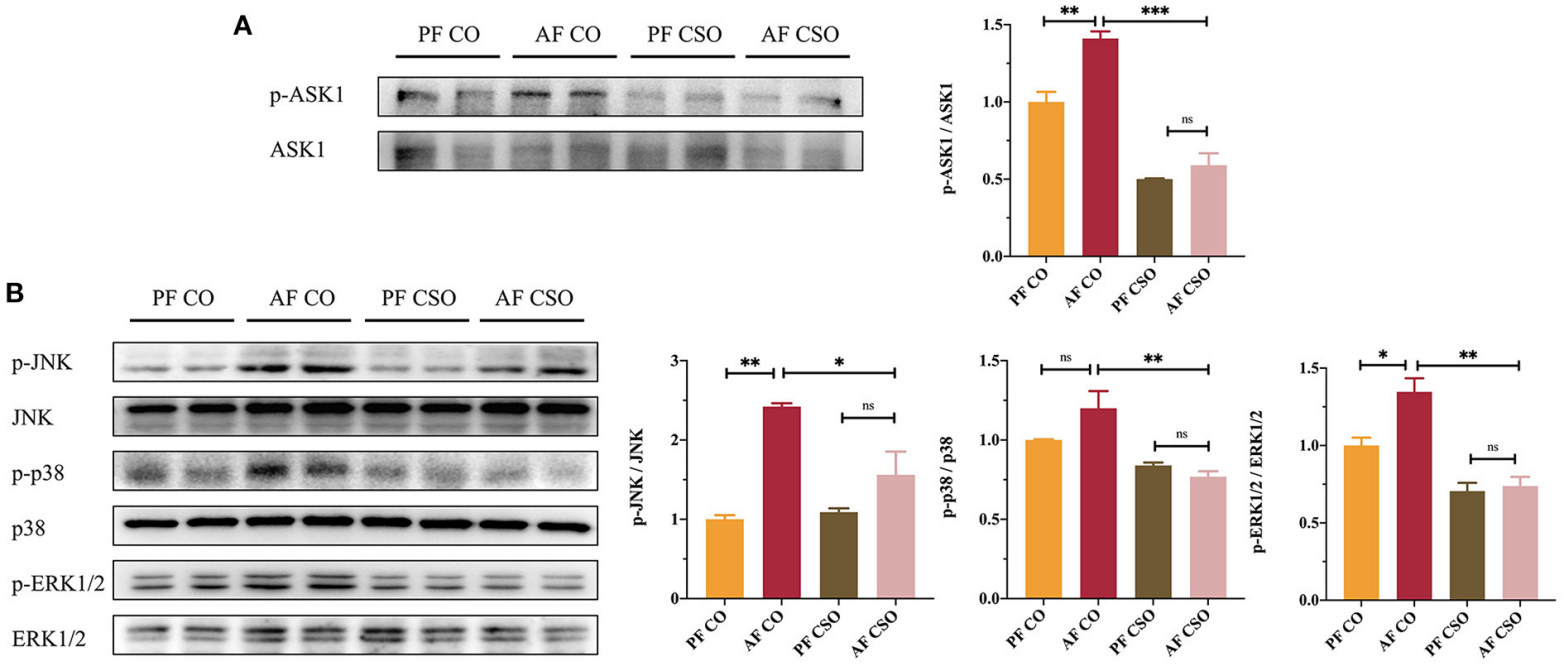
ASK1/MAPKs signaling pathways are involved in CSO-protected liver injury. **(A)** The expression of hepatic p-ASK1/ASK1. **(B)** The expression of hepatic p-JNK/JNK, p-p38/p38, and p-ERK1/2/ERK1/2. *P < 0.05, **P < 0.01, and ***P < 0.001 indicate statistically significant.

We further measured the expression of proteins involved in ASK1 and MAPKs signaling pathways in our study to explore whether CSO regulated ASK1 and MAPKs pathway to alleviate alcohol-induced liver injury. The hepatic protein expressions of p-ASK1/ASK1 (Figure 4A), p-JNK/JNK, p-p38/p38, and p-ERK1/2/ERK1/2 (Figure 4B) among four groups were determined by western-blot, and found that their expressions were significantly reduced in AF/CSO group compared with that in AF/CO group. These data implied that the parallel inhibitions of ASK1 and MAPKs signaling pathways were involved in the action of CSO against alcohol-induced liver injury.

### Dietary CSO supplementation relieves alcohol-induced the imbalance of intestinal flora

**Figure 5:**
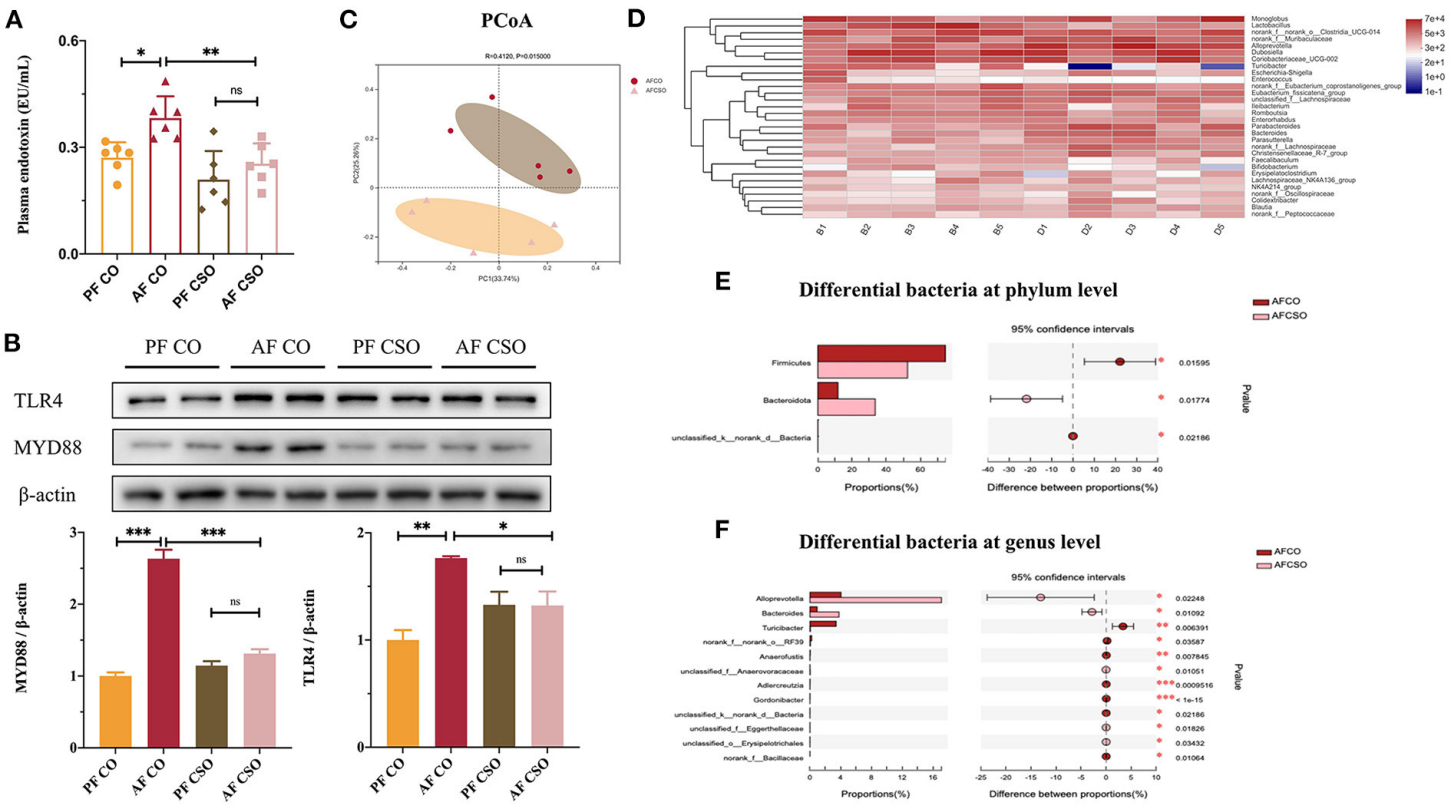
Dietary CSO supplementation relieves alcohol-induced the imbalance of intestinal flora. **(A)** Plasma endotoxin level. **(B)** The expression of hepatic TLR4 and MYD88. **(C)** PCoA score plot. **(D)** Heatmap at genus level. **(E,F)** Kruskal–Wallis *H*-test bar plot on phylum level and genus level. *P < 0.05 and **P < 0.01 indicate statistically significant.

The level of plasma endotoxin was dramatically decreased in AF/CSO group compared with that in AF/CO group (Figure 5A). The expressions of hepatic TLR4 and MYD88 in AF/CSO groups was significantly decreased compared with that in AF/CO group (Figure 5B). PCoA, on the OUT level, of the fecal microbiome diversity revealed an apparent separation between AF/CO and AF/CSO groups (Figure 5C). Besides, the alpha diversity analysis was shown in Table 3. As shown in the community heatmap diagram, the abundance of 30 genera was significantly different between AF/CO and AF/CSO groups (Figure 5D). At the phylum level, the fecal bacterial composition of the AF/CSO group was shifted to a status of lower *Firmicutes* and higher *Bacteroidota* (Figure 5E). At the genus level, *Alloprevotella* and *Bacteroides* were significantly increased, and *Turicibacter* was decreased in AF/CSO group compared with that in AF/CO group (Figure 5F).

**Table 3:** Alpha diversity analysis.

**Estimators**	**AF CO**	**AF CSO**	***P*** **-value**
SOBS	327.800 ± 57.465	312.400 ± 14.011	0.577
CHAO	341.460 ± 57.718	325.060 ± 15.573	0.557
ACE	345.100 ± 60.326	327.690 ± 16.572	0.551
SHANNON	3.201 ± 0.253	3.132 ± 0.330	0.719
SIMPSON	0.090 ± 0.025	0.110 ± 0.037	0.347

CO, corn oil; CSO, camellia seed oil; PF, pair-fed; AF, alcohol-fed.

### Correlation analysis between liver biochemical parameters and the fecal microbiome

**Figure 6:**
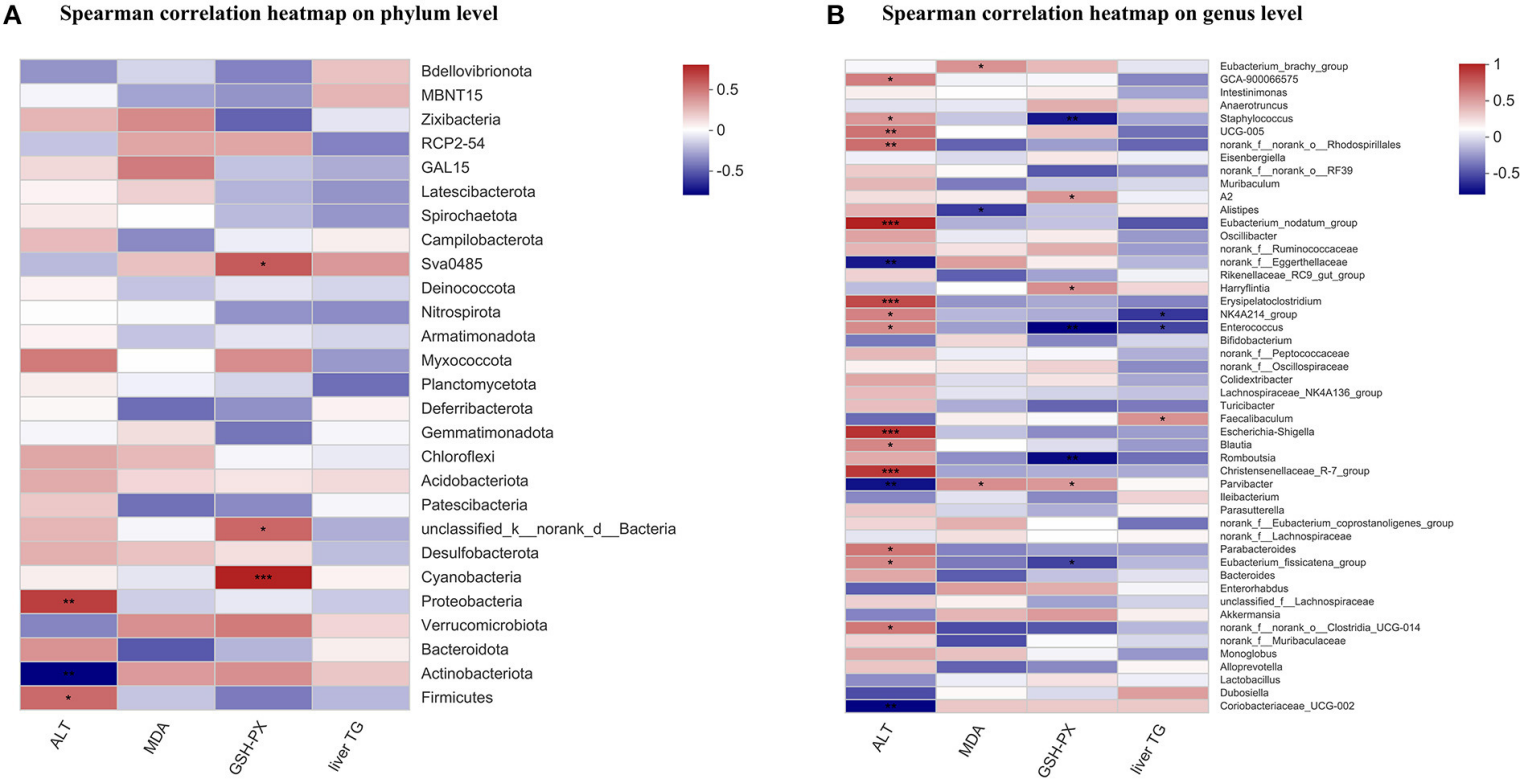
Correlation analysis between liver biochemical parameters and the fecal microbiome. The Spearman's correlation analysis linking the abundance of specific phyla **(A)**, or genera **(B)** with liver biochemical parameters. The Spearman correlation coefficient value was shown in different colors. *P < 0.05, **P < 0.01, and ***P < 0.001 indicate statistically significant.

The Spearman correlation coefficient was calculated in mice to show the effects of plasma ALT, hepatic MDA, hepatic GSH-PX, and liver TG on gut specific bacterial phyla or genera by hierarchical clustering. At the phylum level, the plasma ALT showed a significant positive relation with *Firmicutes* and *Proteobacteria*, and had a significant negative relation with *Actinobacteriota*. The hepatic GSH-PX was positively correlated with *Cyanobacteria, unclassified_k_norank_d_Bacteria*, and *Sva0458* (Figure 6A). At the genus level, the plasma ALT was positively related to 14 bacteria, such as *Christensenellaceae_R-7_group, Escherichia_shigella*, and *Eubacterium_nodatum_group etc*., and was negatively related to three bacteria, including *Coriobacteriaceae_UCG-002, Parvibacter*, and *norank_f_Eggerthellaceae*. The hepatic MDA showed a positive relation with *Parvibacter* and *Eubacterium_brachy_group*, and a negative relation with *Alistipes*. The hepatic GSH-PX was positively related to three bacteria, such as *Parvibacter* and *Harryflintia etc*., and was negatively related to four bacteria, such as *Enterococcus* and *Staphylococcus, etc*. The liver TG had a negative relation with *Enterococcus* and *NK4A214_group*, and a positive relation with *Faecalibaculum* (Figure 6B).

### Dietary CSO supplementation prevents alcohol-induced lipolysis of adipose tissue

**Figure 7:**
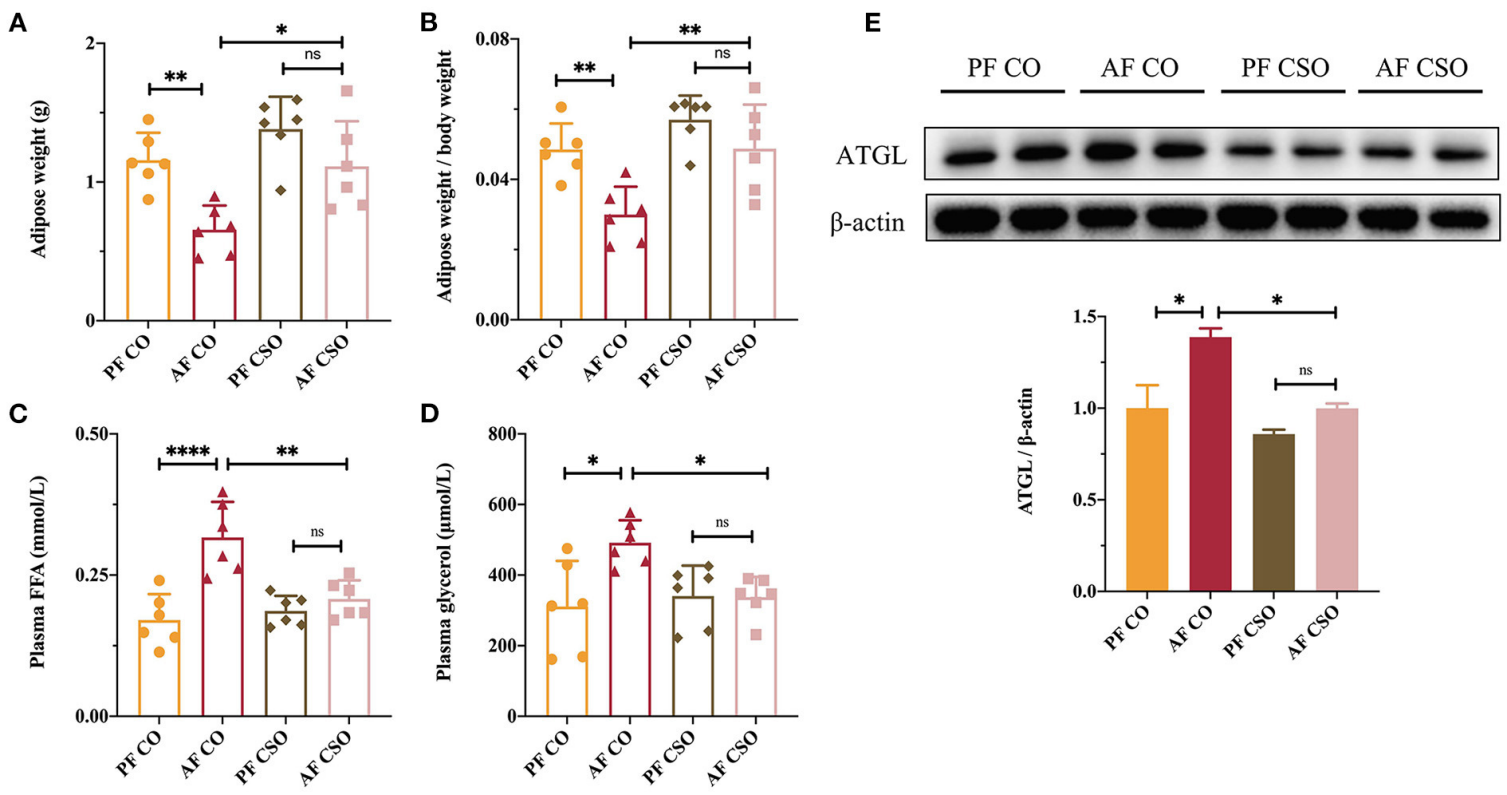
Dietary CSO supplementation prevents alcohol-induced lipolysis of adipose tissue. **(A)** The adipose weight. **(B)** The ratio of adipose-to-body weight. The level of plasma **(C)** FFA and **(D)** glycerol among four groups. **(E)** The expression of ATGL in adipose tissues. *P < 0.05, **P < 0.01, and ****P < 0.0001 indicate statistically significant.

Adipose tissue lipolysis characterized by elevated plasma glycerol and FFA mobilization was thought to aggravate the severity of ALD in the setting of long-term alcohol intervention (18). To investigate mechanism underlying dietary CSO supplementation on alcohol-induced liver injury, we also examined the effect of CSO on adipose tissue lipolysis in ALD mice through the measurement of adipose weight, the ratio of adipose-to-body weight, plasma FFA level, and the circulatory glycerol level. As shown in Figure 7A, the adipose weight in AF/CO group was lower than in PF/CO group and AF/CSO group, but there was no difference between AF/CSO and PF/CSO groups. Similar trends also occurred in the comparison of the ratio of adipose-to-body weight among four groups (Figure 7B). Comparable plasma FFA and glycerol levels were observed between AF/CO and PF/CO groups, and AF/CO and AF/CSO groups, suggesting that dietary CSO could decrease the levels of two adipose tissue lipolysis parameters (Figures 7C,D). Furthermore, we also examined the protein expression of ATGL in adipose tissues of mice by western-blot, and found that ATGL protein was increased in AF/CO group compared with that in PF/CO group or AF/CSO group (Figure 7E). These data implied that dietary CSO supplementation could prevent the adipose tissue lipolysis in ALD mice.

### Dietary CSO supplementation exacerbates alcohol-induced hepatic steatosis

Hepatic steatosis was stained by Oil Red O and photomicrographs from representative mice were shown in Figure S2A. Unexpectedly, compared with CO, the level of hepatic TG was significantly increased by dietary CSO supplementation (Figure S2B). The levels of plasma TG and TC in AF/CO group were clearly increased by 1.56- and 1.37-fold compared with that in PF/CO group, respectively. However, the difference in the levels of hepatic TC, plasma TG, and plasma TC between AF/CO and AF/CSO groups was not statistically significant (Figures S2C–E). The predominant types of fatty acid in CO and CSO are LA (C18:2n-6) and OA (C18:1n-9), respectively. To directly detect the effects of LA and OA on TG level, we assessed their effects via AML-12 cells. The result showed that the level of TG was increased more in purified OA group than in purified LA group under alcohol intervention (Figure S2F).

### Dietary CSO supplementation increases the expressions of hepatic SREBP-1c and DGAT2 in ALD mice

Several key enzymes for lipogenesis, lipolysis, and transport of lipids were detected to investigate the mechanism of dietary CSO supplementation exacerbating alcohol-induced hepatic steatosis. As shown in Figure S3A, the hepatic SREBP-1c protein, which controls the *de novo* synthesis of fatty acids, was obviously activated in AF/CSO group; however, the difference in the protein expressions of CD36 and FATP2 in mice livers between AF/CO and AF/CSO groups was not statistically significant. Meanwhile, the hepatic DGAT2 protein, a rate-limiting enzyme in the regulation of TG synthesis, was also significantly increased in AF/CSO group; however, there were no significant changes in the protein expressions of ATGL, HSL, and VLDLR among four groups (Figure S3B).

## Discussion

In this study, we demonstrated for the first time that MUFAs enriched CSO as unique dietary fat source attenuated chronic alcohol consumption-induced liver injury, hepatic oxidative stress, and inflammation in C57BL/6 mice compared to PUFAs enriched CO (Figures 1–3). Dietary fats, especially the types and proportions of fatty acids, are closely related to the occurrence and development of chronic metabolic diseases, such as cardiovascular disease, obesity, diabetes, and non-alcoholic fatty liver disease etc (19–21). Our previous study found that a saturated fatty acid-rich high-fat diet (HFD) was more detrimental to the liver than an isocaloric unsaturated HFD rich in fatty acids (22). Nowadays, there is no sufficient scientific basis for how dietary fats affect liver-health in people who drink alcohol, but animal experiments including ours found that saturated fatty acids improved chronic alcohol-induced liver injury compared with CO (23, 24). Besides, n-3 PUFAs-enriched oil, such as flaxseed oil, also alleviated chronic alcohol-induced liver dysfunctions via ameliorating lipid homeostasis at adipose tissue-liver axis *in vivo* (25). However, how MUFAs enriched oil supplementation as unique dietary fat source determine the pathological development of ALD is less documented. Therefore, our study was conducted to explore the beneficial effect of CSO, as unique dietary fat source, on ALD *in vivo*. It is worth noting that we chose CSO as the research object for the following reasons: (1) CSO is one of the vegetable oils with the highest content of MUFAs in nature; (2) CSO is the main dietary oil in southern China; (3) Previous studies have shown that dietary CSO has antioxidant, anti-inflammatory, and hepatoprotective bioactivity (3, 10). Actually, multiple plant oils in nature are enriched with MUFAs, including olive oil, canola oil, safflower seed oil and grape seed oil *etc*, and the relationship between those oils and ALD is also worthy of in-depth study.

Accumulating evidence have revealed that generation of hepatic oxidative stress and liver inflammatory responses have a central and causal effect on the pathophysiology of alcohol-induced liver injury (26, 27). Excessive alcohol intake results in the overproduction of reactive oxygen species (ROS), including hydroxyl radical, hydrogen peroxide, and superoxide anion. ROS-mediated oxidative stress is probably the most important reaction involved in alcohol-induced liver injury by the formation of toxic aldehydes, including MDA and 4-HNE, and the destruction of antioxidant systems, such as the decreased GSH-PX level (28). Our data showed that dietary CSO greatly curbed the activity of hepatic MDA, restored the level of hepatic GSH-PX, and decreased the high expression 4-HNE induced by alcohol (Figures 2A–C). Besides, the relationship between HIF-1α and alcohol consumption has been widely examined in mice livers. HIF-1α, a transcription factor, are involved in processes such as oxidative stress, cell proliferation, and apoptosis in *vivo* and *vitro* (29). Both binge and chronic alcohol consumption elevated the expression of HIF-1α in mice livers (30, 31). NRF2 is a master regulator of the intracellular adaptive antioxidant response to oxidative stress and the activation of NRF2 has been extensively demonstrated to provide protection against ALD (32). In our study, compared with CO, dietary CSO obviously decreased the hepatic HIF-1α expression and increased the hepatic NRF2 expression in ALD mice (Figure 2D).

Additionally, chronic alcohol consumption promotes F4/80 positive monocytes infiltration in the liver, alone with more proinflammatory factors, including TNF-α and IL-6, production (33). Our data also showed that dietary CSO with alcohol feeding drastically reduced the F4/80 immunostaining and hepatic TNF-α and IL-6 expressions (Figure 3). Mechanistic investigations further demonstrated that the parallel inhibitions of ASK1 and MAPKs, including JNK, p38, and ERK1/2, were involved in the action of CSO against alcohol-induced liver injury (Figure 4). Inhibition of ASK1 ameliorated chronic-plus-binge alcohol-induced hepatic inflammation and oxidative stress in C57BL/6J mice (34). Several lines of evidence demonstrated that MAPKs signaling pathways could be activated by alcohol exposure in *vivo* and *vitro* (35, 36). Briefly, these data indicated that dietary CSO was better able to ameliorate impairment of the hepatic anti-oxidant and anti-inflammation systems induced by alcohol consumption.

Furthermore, in recent years, the relationship between the intestinal microbiota and ALD has been highlighted. Alcohol abuse not only triggers qualitative and quantitative modifications in intestinal flora taxonomic composition, but also results in the intestinal hypermobility, which leads to the leakage of lipopolysaccharide (LPS) to the bloodstream, further exacerbating the alcohol-induced liver damage (37). Gut microbiota is a complex microbial environment where dynamic mutualistic interactions related to digestion and the absorption of dietary components take place. The consumption of various dietary fat may modulate the gut microbiota composition with effects on host health. In the present study, we found that the level of plasma endotoxin was dramatically decreased in AF/CSO group compared to AF/CO group (Figure 5A), and the expressions of hepatic TLR4 and its adaptor protein-MYD88 was significantly decreased in AF/CSO group compared to AF/CO group (Figure 5B). Bacterial endotoxin, lipopolysaccharide, is a prototypic microbe-derived inflammatory signal that contributes to inflammation in ALD through activation of the TLR4 (38). Meanwhile, the high concentration of plasma endotoxin also derived from the imbalance of gut microbiome (39). Therefore, in order to further evaluate the effect of dietary CSO supplementation on the composition and abundance of the fecal microbiome in ALD mice, 16S rRNA high throughput sequencing analysis was executed. In our study, *Firmicutes* and *Bacteroidota* were the dominant phyla, and *Alloprevotella, Bacteroides*, and *Turicibacter* were the dominant genera, in the gut microbiota in comparison of differential bacteria between AF/CO and AF/CSO groups. Gut dysbiosis with decreased ratio of *Bacteroidota*/*Firmicutes* is reported to be caused by various environmental factors, including alcohol drinking (40). The fecal bacterial composition of the AF/CSO group showed a significant status of lower *Firmicutes* and higher *Bacteroidota* (Figure 5E), suggesting that dietary CSO may restore alcohol-induced intestinal dysbacteriosis with the increased ratio of *Bacteroidota*/*Firmicutes*. Moreover, the Spearman's correlation analysis showed that the plasma ALT had a significant positive correlation with *Firmicutes* (Figure 6A). Reduced ALT activity and low abundance of *Firmicutes*, in our study, indicated that dietary CSO may partially attenuate alcohol-induced liver injury *via* enterohepatic axis.

In addition to the gut microbiota disturbance, the enhancement of adipose tissue lipolysis was thought to another important pathological factor implicating in ALD pathogenesis (41). Inhibition of adipose tissue hydrolysis is one of the important physiological functions of insulin (42). Long-term alcohol consumption can lead to insulin resistance in peripheral tissues, especially adipose tissue, which further promotes lipolysis enhancement and in tune results in an elevation of circulatory FFAs (18). Excessive FFAs uptake by the liver can cause lipotoxicity (43). In our study, the increased adipose weight and the ratio of adipose-to-body weight, and the decreased plasma FFA and glycerol levels in AF/CSO group suggested that dietary CSO supplementation inhibited the adipose tissue lipolysis in ALD mice (Figure 7).

It is of interest that in our study, dietary CSO aggravated lipid accumulation in ALD mice and AML12 cells, evidenced by the Oil Red O stain and the increased hepatic TG content (Figure S2). Further mechanistic investigations showed that the expressions of SREBP-1c and DGAT2 were significantly upregulated in AF/CSO group (Figure S3). The reason for the differential regulation of dietary CSO on hepatic lipid accumulation and injury is unclear so far; however, it is unequivocal that increasing TG synthesis is protective in hepatocytes against non-esterified free fatty acids-induced lipotoxicity (44). Genetically silencing DGAT2, an essential enzyme catalyzing the final step in hepatocyte TG biosynthesis, rescued hepatic steatosis, but aggravated liver injury (45). Similar results were observed in methionine choline-deficient diet-fed mice with IL-6 signaling blockade and CD18 mutation, respectively (46, 47). Moreover, our finding is also in agreement with previous studies, which have shown that CSO pretreatment increased expression of genes related to *de novo* synthesis of fatty acids, such as SREBP-1c, in differentiated bovine mammary epithelial cells (48), and have found that the degree of lipid droplet accumulation in the hepatocytes in the CSO-fed group was lower than that in the olive oil-fed group, but higher than that in the soybean oil-fed group via the cytological observation (49).

**Figure 8:**
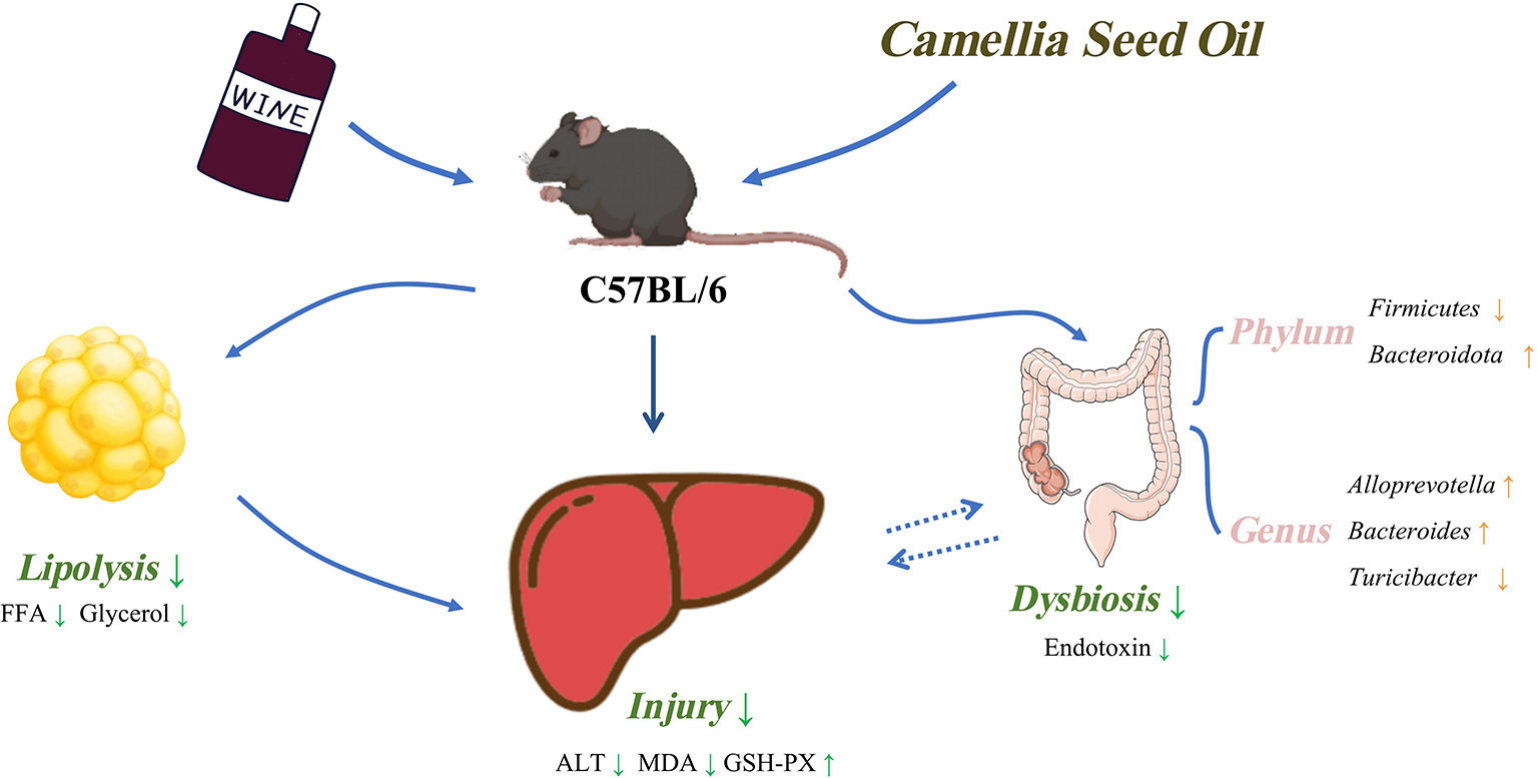
Schematic diagram of this study.

In conclusion, the findings of our study revealed that MUFAs enriched CSO intake exerted beneficial effects on chronic alcohol consumption-induced liver dysfunction in C57BL/6 mice, including improving hepatic oxidative stress and inflammatory response. Dietary CSO also reduced circulating endotoxins, reversed gut microbiota dysbiosis, and prevented adipose tissue lipolysis. Unexpectedly, CSO-feed induced more hepatic steatosis than CO-feed in *vivo* (Figure 8). Results of our study may contribute to understanding the role played by MUFAs enriched CSO in chronic alcohol consumption-induced liver injury and provide a new potential dietary option for alcoholic individuals.

## Data availability statement

The datasets presented in this study can be found in online repositories. The names of the repository/repositories and accession number(s) can be found at: https://www.ncbi.nlm.nih.gov/: PRJNA841488.

## Ethics statement

The animal study was reviewed and approved by Zhejiang Chinese Medical University.

## Author contributions

SL: conceptualization and resources. RG: writing—original draft, data curation, and formal analysis. JZ and LC: data curation, formal analysis, and methodology. JL and QD: data curation and investigation. QH: investigation. WZ: conceptualization. All authors contributed to the article and approved the submitted version.

## Funding

This work was supported by grants from the Zhejiang Natural Science Foundation for Distinguished Young Scholars (LR20H260001), Natural Science Foundation of China (81973041), Special Support Program for High Level Talents in Zhejiang Province (No. ZJWR0308092), and Research Project of Zhejiang Chinese Medical University (2021JKZDZC08).

## Conflict of interest

The authors declare that the research was conducted in the absence of any commercial or financial relationships that could be construed as a potential conflict of interest.

## Publisher's note

All claims expressed in this article are solely those of the authors and do not necessarily represent those of their affiliated organizations, or those of the publisher, the editors and the reviewers. Any product that may be evaluated in this article, or claim that may be made by its manufacturer, is not guaranteed or endorsed by the publisher.

## Abbreviations

4-HNE: 4-hydroxynonenal
ALD: alcoholic liver disease
AF: alcohol-fed
ALT: alanine aminotransferase
ATGL: adipose triglyceride lipase
ASK1: apoptosis signal-regulating kinase-1
BW: body weight
CSO: camellia seed oil
CO: corn oil
CD36: cluster of differentiation-36
DGAT2: diacylglycerol acyltransferase-2
ERK1/2: extracellular signal-regulated kinases-1/2
FFA: free fatty acid
FATP2: fatty acid transport protein-2
GSH-PX: glutathione peroxidase
HSL: hormone-sensitive lipase
HIF-1α: hypoxia inducible factor-1α
IL-6: interleukin-6
IL-1β: interleukin-1β
JNK: c-Jun N-terminal kinase
LA: linoleic acid
MDA: malondialdehyde
MUFA: monounsaturated fatty acid
MYD88: myeloid differentiation factor-88
NRF2: nuclear factor E2-related factor-2
OA: oleic acid
OUT: Operational Taxonomic Units
PF: pair-fed
SREBP-1c: sterol regulatory element binding protein-1c
TG: triglyceride
TC: total cholesterol
TNF-α: tumor necrosis factor α
LW: liver weight
TLR4: toll-like receptor-4
VLDLR: very low-density lipoprotein receptor.
