# Supplementary material for: Dietary camellia seed oil attenuates liver injury in mice chronically exposed to alcohol
Source: Front Nutr. 2022 Oct 11;9:1026740. doi: 10.3389/fnut.2022.1026740 (PMC9598421; doi:10.3389/fnut.2022.1026740)
Supplement: Supplementary file 1 [file Image_1.pdf]

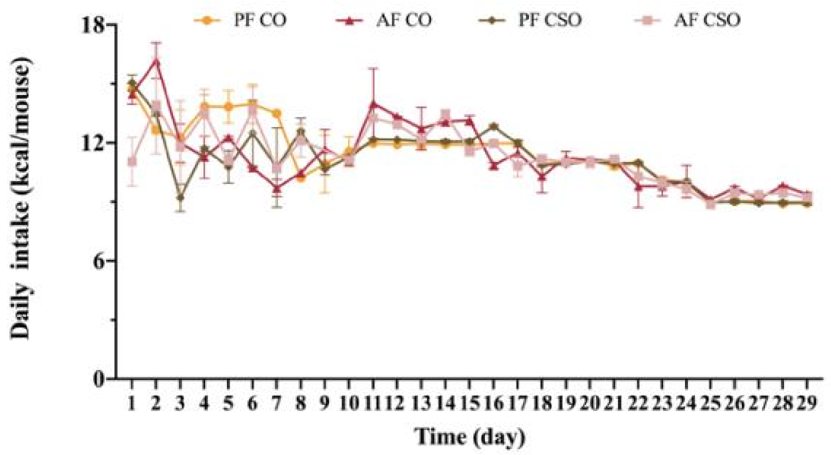

Figure S1. Average daily caloric of liquid intake.

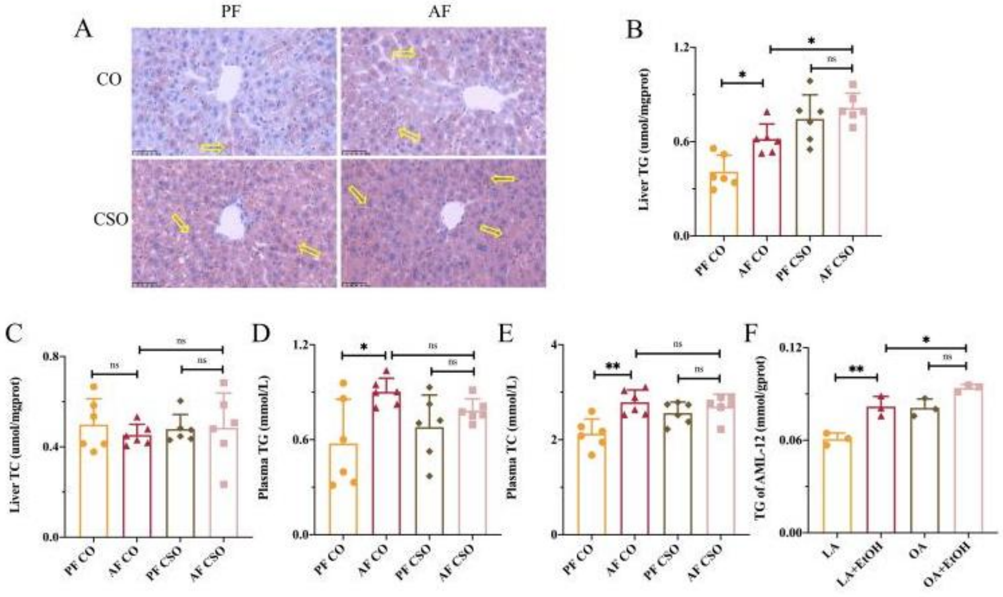

Figure S2. Dietary CSO supplementation exacerbates alcohol-induced hepatic steatosis. (A) Oil red O staining (400 x). The yellow arrow points to lipid droplets. The levels of hepatic (B) TG and (C) TC among four groups. The levels of plasma (D) TG and (E) TC among four groups. (F) The TG content in AML-12 cells induced by LA/OA with/without alcohol (n = 3). EtOH, alcohol. $^{*}p < 0.05$ and $^{**}p < 0.01$ indicate statistically significant.

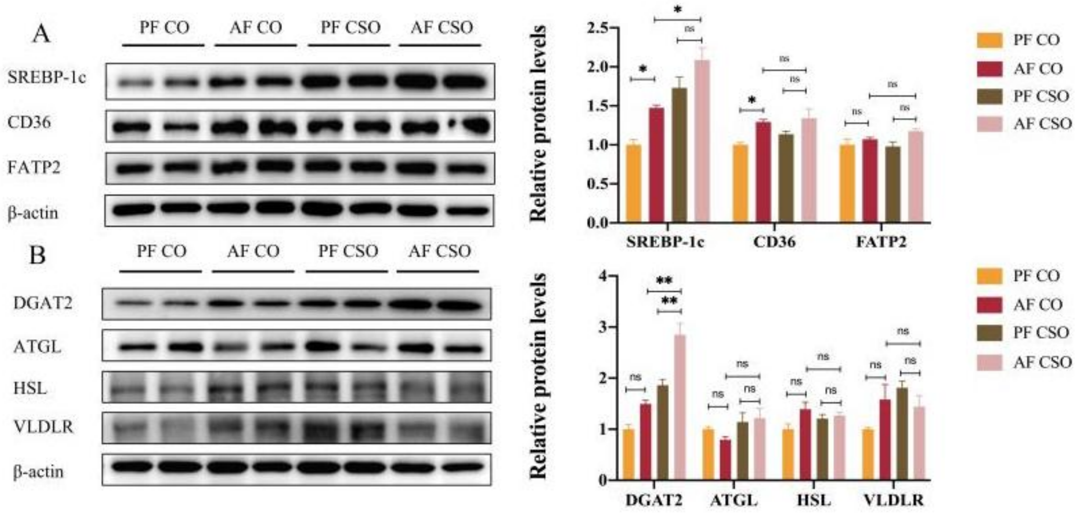

Figure S3. Dietary CSO supplementation increases the expressions of hepatic SREBP-1c and DGAT2 in ALD mice. (A) The expression of hepatic SREBP-1c, CD36, and FATP2. (B) The expression of hepatic DGAT2, ATGL, HSL, and VLDLR. $^{*}p < 0.05$ and $^{**}p < 0.01$ indicate statistically significant.
